# Serum leptin and neuropeptide Y in patients with cystic fibrosis—A single center study

**DOI:** 10.3389/fmed.2022.959584

**Published:** 2022-09-14

**Authors:** Sabina Galiniak, Rafał Podgórski, Marta Rachel, Artur Mazur

**Affiliations:** Institute of Medical Sciences, Medical College, Rzeszow University, Rzeszow, Poland

**Keywords:** body mass index, cystic fibrosis, leptin, neuropeptide Y, FEV_1_ % predicted

## Abstract

Along with the significant elongation in the average life expectancy of patients with cystic fibrosis (CF), there are still significant discrepancies in the height, weight, and body mass index (BMI) of patients compared to controls without CF. The correlation between hormones that regulate appetite and body fat mass may be an additional factor in weight loss or poor weight gain in CF patients. Our objective was to estimate serum concentrations of leptin and neuropeptide Y in patients with CF as well as to assess correlations between studied hormones and the clinical parameters of CF. Leptin and neuropeptide Y serum concentrations after an overnight fast were measured using an enzyme-linked immunosorbent assay. All study participants had anthropometric tests and spirometry. In addition, fasting serum lipid profile was also analyzed. Fasting leptin levels in CF were significantly higher in patients with CF patients (13.9 ± 6.9 vs. 6.5 ± 2.6 ng/mL, *p* < 0.001) compared to controls. There were no differences in leptin concentration between female and male CF participants (15.7 ± 7.8 vs. 12.2 ± 5.6 ng/mL, *p* = 0.13). Leptin was correlated with age (*R* = 0.64, *p* < 0.001), BMI (*R* = 0.65, *p* < 0.001), spirometry results (*R* = −0.49, *p* < 0.01), and body fat (*R* = 0.5, *p* < 0.05). There were no differences in neuropeptide Y concentration between participants with CF and controls as well as neuropeptide Y was not correlated with any studied parameters. The results of our study suggest that weight loss may be associated with a decreased level of leptin, while reduced pulmonary function in CF may be related to an elevated level of leptin.

## Introduction

Cystic fibrosis (CF) is a heterogeneous multiorgan disease caused by mutations in a gene that leads to a defective or missing CF transmembrane conductance regulator. As survival time increases, technological advances and improved quality of health care, there are still significant discrepancies in height, weight, and body mass index (BMI) of patients with CF compared to healthy controls (1, 2). Approximately 70% of CF patients suffer from malnutrition, and in some patients, additionally, body weight deficiency, growth deficiency, reduced brachial fold thickness and reduced lean tissue mass are also observed (3). In addition, fat-soluble vitamin deficiencies are common (4). Due to the fact that the nutritional status of the CF patient is closely correlated with the functioning of the respiratory system, systematic monitoring of the patient and the prevention of nutritional disorder is recommended (5). Leptin is a peptide hormone secreted by adipocytes that regulates food intake, body mass, reproductive functioning and plays a vital role in fetal growth, proinflammatory immune responses, angiogenesis, and lipolysis (6). Leptin levels may reflect the body fat levels. It is known to be increased in obese people and decreased in lean people (7).

On the other hand, neuropeptide Y is peptide hormone found in the brain which stimulates food intake, decreases thermogenesis, and increases plasma insulin and corticosterone levels making it a potential target. Leptin is known to regulate appetite and metabolism by inhibiting the synthesis and release of neuropeptide Y in the arcuate nucleus (8). However, current data on circulating leptin and neuropeptide Y levels in CF are inconsistent and indicate decreased, increased, or unchanged levels of these hormones (9–11). Additionally, leptin and neuropeptide Y levels may correlate with patients' clinical parameters, including age and body fat (9, 11). The altered hormone levels in CF compared to controls could be an explanation of the decreased appetite and the poor weight gain observed in CF patients. Therefore, our objective was to estimate serum leptin and neuropeptide Y concentrations in patients with CF, as well as to assess any correlations between leptin and neuropeptide Y and the clinical parameters of CF.

## Materials and methods

### Ethics approval

Bioethics Committee of the Rzeszów University approved the study design (2022/023). All procedures performed in studies involving human participants were in accordance with the ethical standards of the institutional and/or national research committee and with the Declaration of Helsinki of 1964 and its subsequent amendments or comparable ethical standards. All patients and/or legal guardians provided written informed consent.

### Study design and study sample

A single-center, cross-sectional study was carried out in a group of 38 CF patients and 16 controls. Participants were recruited from the local CF center in Provincial Hospital No. 2, Rzeszow, Poland, between 02/2021 and 10/2021.

Patients with CF had a confirmed diagnosis based on the measurement of chloride in sweat, genetic testing, and immunoreactive trypsin test in neonatal age (patients born in or after 2009). Subsequent criteria for involving patients in the study were as follows: forced expiratory volume in the first second (FEV_1_) more than 35% of predicted stable pulmonary disease and no hospital stays in the 30 days before screening. Exclusion criteria included: heart and liver dysfunction, psychiatric disorder, post-solid organ transplantation and corticosteroids treatment. Moreover, inability to perform spirometry and refusal to participate in the study were exclusion criteria. Due to these reasons, 9 patients were excluded from the study (4 patients refused to participate in the study, 3 patients had a lung transplant, and 2 were unable to perform spirometry). All CF patients suffered from pancreatic insufficiency and received treatment as recommended (Creon 25000, Solvay Pharmaceutical Inc., Marietta, Georgia, USA; Pulmozyme, Genentech Inc., San Francisco, California, USA; one 2.5 mg ampoule inhaled once daily using a nebulizer; ADEK tablets, Scandipharm, Birmingham, Alabama, USA; nutrition drinks, Nutrison Protein Plus, Nutricia, Poland; inhalation of 3–10% sodium chloride 3–4 times daily).

Healthy subjects were included in the study from the local clinic. The control group consisted of age- and sex-matched participants who had no diseases in medical history or physical examination. The healthy participants had not taken any drugs and supplements 30 days prior to the study. All participants had anthropometric measurements. BMI was calculated as kg/m^2^. Percent of fat and fat mass were estimated by using calibrated digital scale Tanita MC980MA^+^ (Tokio, Japan).

### Spirometry

All participants had a spirometry test (Lungtest 1000, MES, Kraków, Poland) according to recommendations (12). We calculated the mean value of the last 6 months for forced vital capacity (FVC), FEV_1_, FEV_1_/FVC and expressed as a percentage of the predicted value for age and sex.

### Blood sampling

Blood samples were obtained, centrifuged, aliquoted and storage according to standard procedure.

### Blood counts and serum analysis

Blood morphology was performed by using standard analyzer (Siemens Healthineers, Germany). Serum lipid profile was estimated by using enzymatic methods and expressed as mg/dL.

### Leptin

Leptin serum level after an overnight fast were measured in duplicates using the enzyme-linked immunosorbent assay (Wuhan Fine Biotech Co., Ltd., Wuhan, China), according to the protocol and the results were presented as nanograms per milliliter.

### Neuropeptide Y

Neuropeptide Y serum concentrations after an overnight fast were measured in duplicates using the enzyme-linked immunosorbent assay (Wuhan Fine Biotech Co., Ltd., Wuhan, China), according to the protocol and the results were presented as picograms per milliliter.

### Statistical analysis

STATISTICA software package (version 13.3, StatSoft Inc. 2017, Tulsa, OK, USA) was used for statistical analysis of the results. Data are shown as mean ± SD and range. Comparisons of the groups were performed with the Mann-Whitney *U*-test or Kruskal-Wallis test due to the lack of a normal distribution of the results. Normality was verified using the Shapiro Wilk normality test. The associations between results was performed using Spearman's rank correlation coefficient analysis, assuming linear dependence with 95% confidence interval.

## Results

Study included a total of 17 females and 21 males with CF. Moreover, 10 healthy females and 6 males were enrolled into the control group. Baseline characteristics, clinical hematology values, and indices of pulmonary function for patients with CF and healthy volunteers are presented in Table 1.

**Table 1:** Demographic data of the participants at enrolment^*^.

		**CF**	**Healthy controls**	***p***
Sex (F/M)		17/21	10/6	
Age (years)	Mean ± SD	19.58 ± 7.9	19.25 ± 7.3	0.855
	Range	10–39	10–38	
Height (cm)	Mean ± SD	157.56 ± 18.1	160.56 ± 15.1	0.598
	Range	124–188.5	130–180	
Weight (kg)	Mean ± SD	50.03 ± 13	58.75 ± 13.9	**0.038**
	Range	22.1–76	34–82	
BMI (kg/m^2^)	Mean ± SD	19.89 ± 2.8	22.47 ± 2.5	**0.003**
	Range	14.4–25.9	18.7–25.6	
Body fat (%)	Mean ± SD	19.24 ± 5.05	18.46 ± 3.45	0.353
	Range	7.9–25	14–24	
Fat mass (kg)	Mean ± SD	9.86 ± 2.82	10.86 ± 2.25	0.241
	Range	5.2–14.6	6.8–14	
**Genotype**				
Homozygous ΔF508, n (%)		30 (78.9)	–	–
Heterozygous ΔF508, n (%)		8 (21.1)	–	–
**Clinical laboratory markers**				
WBC (10^3^/μL)	Mean ± SD	9.95 ± 3.6	7.46 ± 2.3	**0.022**
	Range	5.1–19.3	4.3–10.5	
NEU (%)	Mean ± SD	61.01 ± 15.3	59.12 ± 6.1	0.605
	Range	25.1–82.3	50.6–68.6	
**Lipid profile**				
Cholesterol (mg/dL)	Mean ± SD	121.04 ± 22.58	100.81 ± 13.29	**0.001**
	Range	79–191	85–125	
HDL in males (mg/dL)	Mean ± SD	43.19 ± 9.93	45.83 ± 5	0.494
	Range	26–66	42–51	
HDL in females (mg/dL)	Mean ± SD	41.17 ± 5.47	45.4 ± 2.99	**0.025**
	Range	34–53	42–54	
LDL (mg/dL)	Mean ± SD	65.43 ± 15.7	78.25 ± 16.99	**0.015**
	Range	41–108	45–110	
Triglycerides (mg/dL)	Mean ± SD	85.28 ± 35.73	86.69 ± 12.04	0.12
	Range	42–172	68–101	
**Pulmonary function**				
FEV_1_ (% predicted)	Mean ± SD	86.35 ± 27	102.4 ± 8.2	**0.006**
	Range	35–142	97–127	
FVC (% predicted)	Mean ± SD	93.5 ± 22.3	103.4 ± 5.8	**0.001**
	Range	52–142	98–118	
FEV_1_/FVC (% predicted)	Mean ± SD	88.82 ± 18	100.7 ± 5.7	**0.001**
	Range	35–120	94–120	

* BMI, body mass index; WBC, white blood cells; NEU, neutrophils; HDL, high-density lipoprotein; LDL, low-density lipoprotein; FEV_1_, forced expiratory volume in 1 s; FVC, forced vital capacity; data are presented as mean ± SD and median (interquartile range) for CF and control. Statistically significant differences are in bold.

There were no differences in age and height between patient with CF and healthy controls. Weight and BMI were significantly lower, whereas the percent of fat and fat mass were similar in studied groups. Among CF patients 30 were homozygous while 8 were heterozygous for ΔF508. Levels of white blood cell were significantly elevated in subjects with CF as compared with controls. Concentration of cholesterol was higher, while LDL was lower in CF than healthy volunteers. Level of HDL was similar in males with CF and healthy males, whereas it was decreased in females with CF as compared to control females (*p* < 0.05). Pulmonary function indices were significantly worse in CF participants (*p* < 0.01).

**Figure 1:**
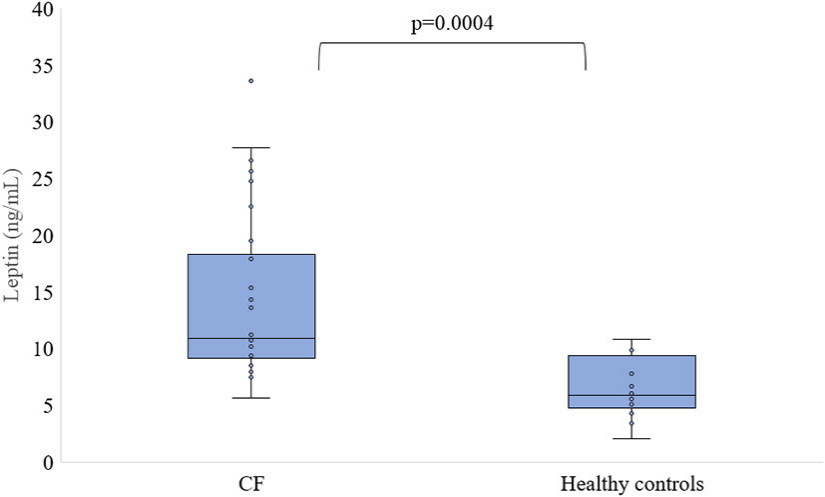
Level of leptin in serum of patients with CF and healthy controls.

Fasting leptin levels in CF were significantly higher in patients with CF patients (13.9 ± 6.9 vs. 6.5 ± 2.6 ng/mL, *p* < 0.001) compared to controls (Figure 1).

**Figure 2:**
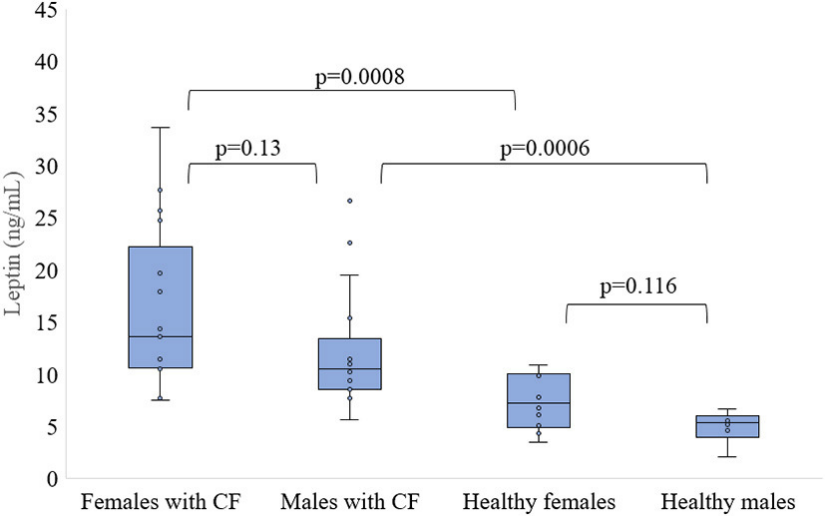
Level of leptin in serum of patients with CF and healthy controls depending of sex.

There were no differences in leptin concentration between female and male CF participants (15.78 ± 7.85 vs. 12.17 ± 5.65 ng/mL, *p* = 0.13) as well as healthy females and males (7.43 ± 2.7 vs. 4.97 ± 1.58 ng/mL, *p* = 0.116). Both females and males with CF had higher leptin levels than respectively healthy females and males (*p* < 0.001, Figure 2).

**Figure 3:**
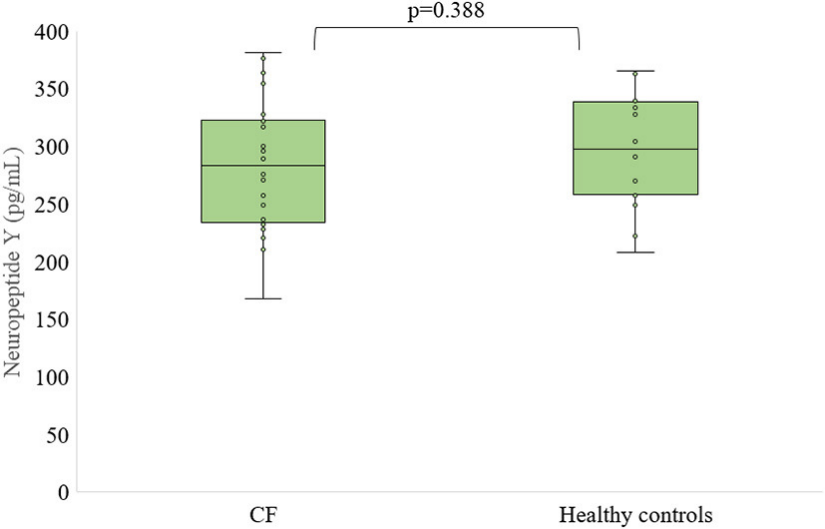
Level of neuropeptide Y in serum of patients with CF and healthy controls.

**Figure 4:**
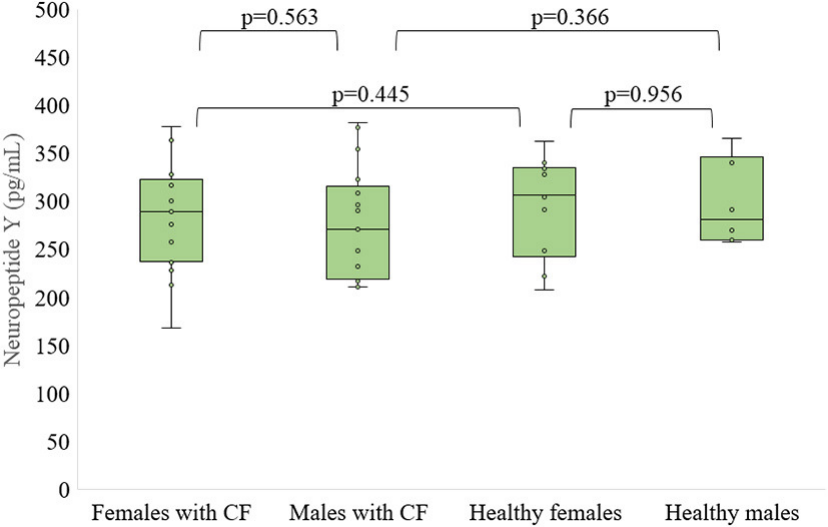
Level of neuropeptide Y in serum of patients with CF and healthy controls depending of sex.

There were no differences in neuropeptide Y concentration between patients with CF and control subjects as well as between females and males who are suffered from CF and healthy, respectively (Figures 3, 4).

We did not find correlation between serum concentration of leptin and neuropeptide Y (*R* = −0.2165, *p* = 0.2187).

Table 2 presents the correlation of leptin and neuropeptide with the results of anthropometric measurements, body fat content, hematological results, lipid profile and lung function indices of the studied patients.

**Table 2:** Correlation coefficients between leptin and neuropeptide Y concentration and clinical parameters of studied patients^*^.

		**Leptin**	**Neuropeptide Y**
Age	*R*	**0.643**	−0.0708
	*p*	**0.0004**	0.6728
Height	*R*	0.002	−0.0217
	*p*	0.99	0.8972
Weight	*R*	**0.344**	−0.0561
	*p*	**0.046**	0.7378
BMI	*R*	**0.649**	−0.1741
	*p*	**0.0002**	0.2959
Body fat (%)	*R*	**0.5011**	0.0045
	*p*	**0.0175**	0.9841
Fat mass	*R*	**0.5025**	0.472
	*p*	**0.0181**	0.0565
WBC	*R*	−0.071	0.2132
	*p*	0.691	0.1986
NEU	*R*	−0.048	0.1253
	*p*	0.786	0.4535
Cholesterol	*R*	0.3462	−0.1218
	*p*	0.1056	0.539
HDL	*R*	0.3108	−0.2533
	*p*	0.1393	0.2217
LDL	*R*	0.24	−0.0842
	*p*	0.2586	0.6889
Triglycerides	*R*	0.136	−0.1527
	*p*	0.5169	0.4565
FEV_1_	*R*	–**0.494**	0.2737
	*p*	**0.003**	0.0962

* BMI, body mass index; WBC, white blood cells; NEU, neutrophils; HDL, high density lipoprotein; LDL, low density lipoprotein; FEV_1_, forced expiratory volume in 1 s. Statistically significant differences are in bold.

**Figure 5:**
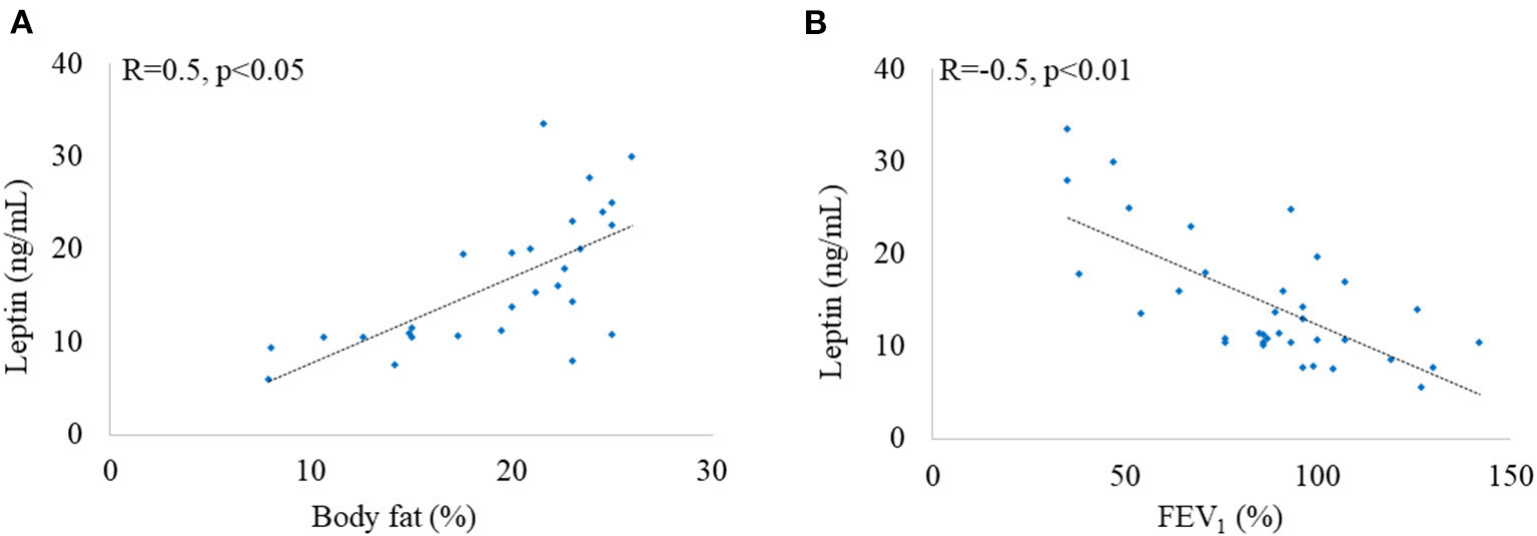
Correlation between leptin and body fat **(A)** and leptin and FEV_1_ **(B)**.

We found a positive significant correlation of leptin with age (*R* = 0.64, *p* < 0.001), weight (*R* = 0.344, *p* < 0.05), and BMI (*R* = 0.65, *p* < 0.001). Moreover, leptin positively correlated with percent of body fat as well as fat mass in patients with CF (Figure 5A). Leptin was negatively correlated with FEV_1_ (*R* = −0.49, *p* < 0.01, Figure 5B).

Leptin was not related with white blood cells, neutrophils and lipid profile. Similarly, neuropeptide Y was not correlated with any studied parameters.

## Discussion

Leptin concentrations reflect the amount of body fat that may be decreased in anorexia or increased in obesity (6, 13). In the literature there are inconsistent data regarding the leptin concentration CF patients, which indicates that there is dysregulation of leptin synthesis among CF patients. Studies in mice model of CF revealed increasing caloric intake while disrupting a metabolic regulatory system, leptin signaling, led to enhanced growth and significant fat stores (14). Furthermore, leptin disturbances also contribute to abnormal immune response as well as susceptibility to infections that are common in CF (15). It is worth mentioning that leptin plays an important role in the development and regulation of the redox system hence elevated leptin levels may induce generation of reactive oxygen species and promote inflammation which may be one of the causes of the exaggerated oxidative stress in CF (16, 17).

Similarly to our results, Stylianou et al. also reported significantly higher leptin level in CF adolescents than healthy controls (*p* = 0.03), as well as elevated hormone level in CF females than CF males (9). Moreover, a higher level of leptin was observed among pediatric CF patients (18, 19). On the other hand, serum leptin concentration was similar in CF and controls (5.3 ± 4.1 vs. 4.4±3.6 ng/mL) in the study by Arumugam et al. (20). However, they also observed a significantly lower leptin level in CF males than females. Nevertheless, decreased leptin concentration was noted in severe CF patients (FEV_1_ < 45%) compared to controls and other CF subjects (10). Likewise, pediatric and youth patients with CF had lower leptin levels compared to the control group (21–23). Contrary to our results, the concentration of leptin in patients below 16 years of age was higher than in older patients indicating that different CF populations may have different leptin concentrations (11). A higher proportion of body fat and an increased rate of leptin synthesis per unit mass of adipose tissue may explain the higher level of serum leptin in women than in men (24). Moreover, elevated leptin levels may be associated with depression and anxiety experienced by CF patients, requiring further investigation and multivariate analysis (25–27).

Neuropeptide Y was of interest in CF due to its close location to CFTR on human chromosome 7, however there is little data on the involvement of this peptide in the pathogenesis of CF (28). A study on 56 patients with CF revealed that the serum neuropeptide Y concentration was 3.7 ± 2.99 ng/mL, while in our study it was 278.9 ± 56 pg/mL (11). Elevated neuropeptide Y was found in olfactory epithelium of CFTR knockout mice (29). It is assumed that neuropeptide Y could reduce antimicrobial secretion or promote inflammation in patients' lungs (30). In our study, there was no difference in neuropeptide Y levels between females and males with CF as well as healthy participants. The statistically non-significant difference between the levels of neuropeptide Y between men and women was described in the study by Nyström et al. (31).

Contrary to our results, serum leptin and neuropeptide Y correlated negatively in study by Nowak et al. (11). After exposure, leptin may rapidly inhibit food intake by lowering the secretion of hypothalamic neuropeptide Y (32).

In normal condition, aging is associated with decreased leptin uptake in the hypothalamus due to reduced expression of leptin receptor mRNA and reduced levels of leptin receptor protein in the hypothalamus (33). This is in contrast to our results which indicate that leptin levels increase with age in CF. The current study demonstrates a positive association between weight, BMI and leptin concentrations as previously described (10, 18, 21). We found no correlations between leptin and patients' height which is in line with a recent study in participants without CF (34). Moreover, leptin concentration was positively correlated with body fat which is in line with study by Stylianou et al. and Grandos et al. (9, 21). The concentration of leptin was positively correlated with anthropometric indicators, fasting glucose, triglycerides, and negatively related with the concentration of HDL and cholesterol in study among 34 patients aged from 3 months to 18 years (35). Nevertheless, we did not find any correlations between leptin and lipid profile in CF. Nonetheless, we found a negative correlation between FEV_1_ and leptin concentration which is different from that described earlier in CF patients aged 22–48 years (10). A study on human coronary artery smooth cells showed that hypoxia increased leptin expression, with earlier expression of angiotensin II and reactive oxygen species (36). A negative correlation between FEV_1_ and serum leptin has also been reported in healthy obese and non-obese children, adults, and asthmatic individuals (37–39). Due to the prolongation of life of patients with CF, more and more comorbidities appear, including cystic fibrosis-related diabetes (CFRD) (40). Disturbance in leptin levels in CFRD patients with lower insulin secretion, may present an early symptom of a cascade for metabolic alternations that may finally lead to negative clinical outcomes in subjects with CF (21). We found no correlation between the level of leptin and the level of white blood cells in the blood and the percentage of neutrophils, indicating that these are independent factors in CF. Nevertheless, white blood count was positively correlated with fasting plasma leptin concentration (*R* = 0.38, *p* = 0.0001) in obese people, which indicate that count of white blood cells to percent body fat may be mediated, in part, through the effect of leptin (41).

In this study, the neuropeptide did not correlate with patients' anthropological, functional and biochemical parameters. This indicates that these factors are not predictors of serum levels of neuropeptide Y in CF patients. Nonetheless, the concentration of neuropeptide Y positively correlated with the age of the patients as previously described (*R* = 0.43, *p* = 0.001) (11). There were no correlations between neuropeptide Y and BMI, waist/hip ratio, blood pressure in study by Nyström et al. Nevertheless, total cholesterol (*R* = 0.39, *p* < 0.0001) and LDL-cholesterol (*R* = 0.35, *p* = 0.0001) were positively correlated with plasma neuropeptide Y in women only (31).

The results of our study suggest that weight loss may be associated with a decreased level of leptin. On the other hand reduced pulmonary function in CF may be related to an elevated level of leptin. These results could provide an explanation and could also be an additional cause of the appetite suppression and poor weight gain seen in CF patients (42).

Interesting and new results are described by us in this study, however, a few limitations should be mentioned. Authors suggested that direct comparisons of reports may be difficult due to the small number of patients as well as the origins of the CF population from different geographic regions and ethnicities. Moreover, most reports of hormone levels in CF have been studied in children and adolescents, and there are actually very few reports on adults, so there may be discrepancies in the results between studies. It should be borne in mind that our study involves a small number of patients, therefore, as mentioned, caution is required in interpreting and comparing the results. Nevertheless, it seems that in the future a multicenter study involving a large group of patients should be conducted to determine concentration of leptin and neuropeptide Y and their influence on the clinical outcomes of patients with CF.

## Data availability statement

The raw data supporting the conclusions of this article will be made available by the authors, without undue reservation.

## Ethics statement

The studies involving human participants were reviewed and approved by Bioethics Committee of the Rzeszów University. Written informed consent to participate in this study was provided by the participants' legal guardian/next of kin.

## Author contributions

SG and AM: conceptualization. SG and RP: data curation, investigation, and methodology. SG: formal analysis, project administration, software, supervision, and writing—original draft. SG and MR: resources. SG, RP, MR, and AM: writing—review and editing. All authors contributed to the article and approved the submitted version.

## Conflict of interest

The authors declare that the research was conducted in the absence of any commercial or financial relationships that could be construed as a potential conflict of interest.

## Publisher's note

All claims expressed in this article are solely those of the authors and do not necessarily represent those of their affiliated organizations, or those of the publisher, the editors and the reviewers. Any product that may be evaluated in this article, or claim that may be made by its manufacturer, is not guaranteed or endorsed by the publisher.
